# Extensive cerebral Sinovenous Thrombosis in a 5 Year Old Girl, Following Mild Dehydration. (Case Report and Review of Literature)

**DOI:** 10.5812/ircmj.6418

**Published:** 2013-12-05

**Authors:** Farah Ashrafzadeh, Javad Akhondian, Mehran Beiraghi Toosi, Nargess Hashemi

**Affiliations:** 1 Department of Pediatric Neurology, Mashhad University of Medical Sciences, Mashhad, IR Iran; 2 Department of Pediatrics, Mashhad University of Medical Sciences, Mashhad, IR Iran

**Keywords:** Cerebrum, Thrombosis, Dehydration, Child

## Abstract

Abstract

Cerebral sinovenous thrombosis (CSVT) in children has rarely been reported in the literature, especially without underlying disorder. It has increasingly been diagnosed due to clinical awareness and sensitive neuroimaging techniques. The aim of this article was to report a case of cerebral sinovenous thrombosis without underlying disorder. We reported a 5 year old girl, presented with severe headache and seizure. She had a history of fever and diarrhea before the onset of headache. Neuroimaging showed evidence of CSVT on MRI and magnetic resonance venography. Investigations showed no inherited thrombophilia. The patient was treated with low molecular weight heparin (LMWH) which continued by warfarin. This case illustrated severe complications of dehydration in pediatrics without any evidence of underlying disorders.

## 1. Introduction

Cerebral sinovenous thrombosis (CSVT) is an uncommon but important diagnosis, as it is remediable when promptly recognized and treated (1). The estimated incidence of pediatric CSVT is 0.5 – 2.7 per 100000 children per year, which more than 40 percent happen in neonates (2). The clinical presentations of CSVT may progressively develop over days or weeks. Most infants and children present with headache, seizure and altered mental status. At the onset of the disease, pseudotumor cerebri is common (3, 4). Hypercoagulant states are frequently associated with childhood venous thrombosis, including CSVT. Drugs, prothrombotic state and common childhood illnesses, including iron deficiency anemia and severe dehydration have been reported in CSVT (3).

Radiologic evaluation of CSVT requires CT scan with contrast enhancement to indicate filling defects in the cerebral venous system, indicating the empty delta sign (2, 5). Because even contrast CT scan frequently misses the diagnosis of CSVT (2, 6), dedicated imaging of the cerebral venous system is needed, including computed tomography venography (CTV) or magnetic resonance venography (MRV) (6, 7). Clinical outcomes include death in 9 - 29 percent and neurological impairments, psychological disorders, and headache in over a half of the survivors (2). Anticoagulant therapy, such as unfractionated heparin or LMWH has an important role in childhood CSVT treatment. In children, Anticoagulant therapy is continued for 3 months. At that time, if the patient achieves recanalization, the treatment is usually discontinued and if not, administration of a further 3 month anticoagulation is recommended (3, 8). Treatments also include hydration, antibiotic therapy, and / or surgery for foci of cranial infection, measures aimed to decreasing intracranial pressure, and anticonvulsants for seizures (8). An association between mild dehydration and CSVT has rarely been reported in the literature. We presented here a 5 year old girl with CSVT following mild dehydration due to diarrhea and high fever.

## 2. Case Presentation

A 5-year-old girl presented with gradually onset of headache and vomiting which was deteriorating over 4 days. The severity of headache and improving this symptom in 3 days was measured by qualitative scale as the patient and her mother said. She had a history of fever and diarrhea one week before the admission. She was admitted to the department of pediatric neurology, Qaem hospital, Mashhad, Iran on January 2012. On the first day of admission, she had a generalized tonic clonic seizure which lasted 5 minutes, and was controlled with diazepam. She had no history of drug consumption or thrombosis and other disorders in herself or her family. On clinical examination she was conscious, agitated and febrile. She had tachycardia (HR: 120 per minute), with a blood pressure of 90 / 50 mm Hg, which was in the normal limit according to the sex and age. She had neck stiffness but no kerning or brudzinski signs. Papilledema was diagnosed by direct ophthalmoscope. On fundoscopic examination, her pupils were equal and reactive to the light, and on the third day of admission she had bilateral papilledema. In direct ophthalmoscopy, the pulse of the ophthalmic central vein was diminished and loss of major vessels as they leave the disc was seen (grade 3 of papilledema).

In the laboratory investigations, apart from leukocytosis (WBC: 30000 & PMN: 80), no abnormality was distinguished in the hematologic profile, liver and renal functions. Serum electrolytes, ECG and Chest X- ray had normal findings. Coagulation profiles were all within the normal limits. The biochemical markers were measured by auto analyzer (Prestige automated analyzer), and CBC (with diff) by Sysmex KX21 hematology analyzer in reference laboratory. Coagulative proteins were measured by Cylex instrument by magnetic method.

**Figure 1.**
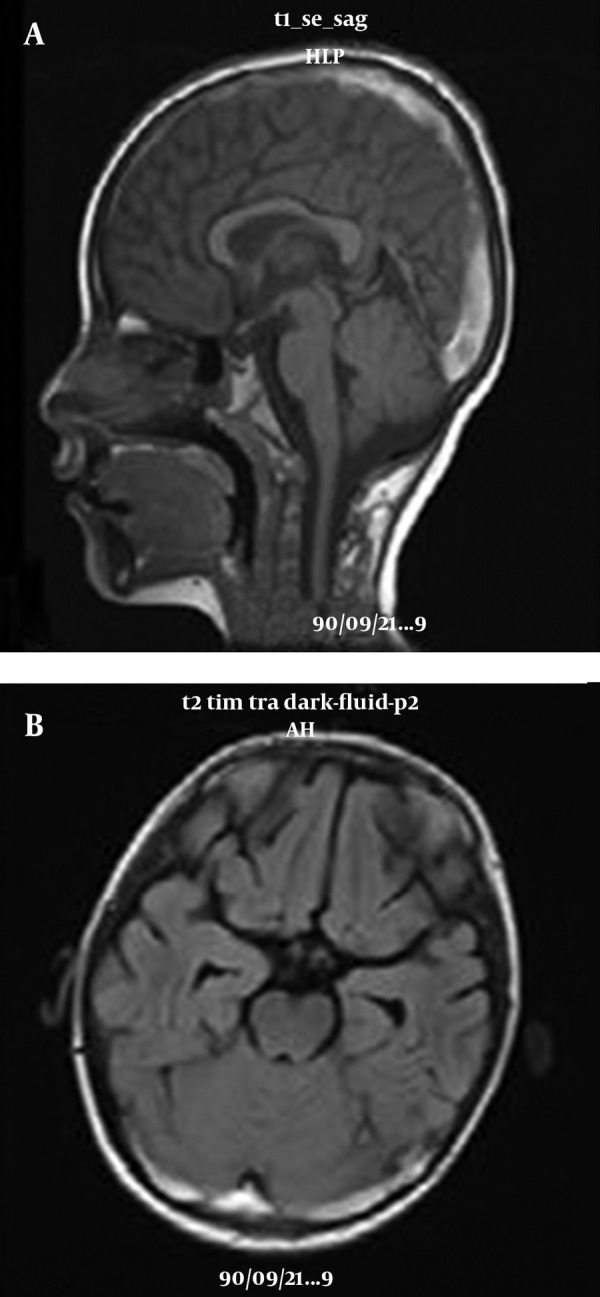
(A), T1-weighted image of brain MRI showed hyper signal intensities in superior sagittal sinus which revealed thrombosis. (B), FLAIR axial MRI showed thrombosis in transverse sinovenouses.

Lumbar puncture was performed and meningitis was ruled out. Echocardiography had normal findings except for a mild pulmonary insufficiency. All the instruments were calibrated and rechecked. CT scan confirmed the presence of thrombosis in the transverse sinovenouses. MRV demonstrated a loss of flow in the superior sagittal, straight and transverse sinovenouses compatible with the diagnosis of CSVT. Figure 1 and T2-weighted images, DWI and ADC map of brain MRI were performed by Siemens MRI scanner (1.5 T) in Ghaem MRI center and were explained by professor Nekoi (radiologist). According to the diagnosis of CSVT the treatment with heparin was begun in the therapeutic dosage. Dexamethasone was used to decrease intracranial pressure and phenytoin for seizure. Because of severe headache due to pseudotumor cerebri, lumbar puncture was performed three times during hospitalization which significantly improved headaches. The screening investigations for inherited thrombophilia including protein C, protein S and antithrombin III deficiencies, factor V Leiden mutation, antiphospholipid antibodies (anticardiolipin IgM and IgG), and lupus anticoagulant had all negative results (Table 1). 

**Table 1:** Coagulation Profile

Parameter	Result	Normal Range
**PT (sec)**	11	10.6 - 11.4
**aPTT (sec)**	32	24 - 36
**INR**	1	0.96 - 1.04
**Pr S (u/mL)**	0.7	0.54 - 1.18
**Pr C (u/mL)**	0.63	0.4 - 0.92
**Antithrombin (u/mL)**	1.29	0.82 - 1.39
**Factor Leiden (u/mL)**	1.1	0.79 - 1.27

After 5 days of treatment she improved significantly. Treatment was begun with warfarin and continued to maintain INR between 2.5 and 3.5. She was discharged on the 14th day after starting the treatment warfarin for 3 months. After 2 weeks, brain CT scan showed no evidence of recanalization but she was symptom free. At follow up one month later, she did not have any neurological deficits.

## 3. Discussion

Heler et al. in a multicenter study, including 149 pediatric patients reported that cerebral sinovenous thrombosis (CSVT) in most cases resulted from prothrombotic risk factors (like factor V Leiden, protein C or S deficiency) and /or underlying clinical conditions (vasculitis, neoplastic disease, trauma, fever, anemia, etc.) (9). Their data were like Veber study who included 92 children with arterial ischemic stroke and sinovenous thrombosis caused mostly (38%) by prothrombotic conditions in particular anticardiolipin antibody disease (33%) (10), unlike adults (11).

There are a few case reports of CSVT during diabetic ketoacidosis (12), with iron deficiency anemia (13), and with idiopathic nephrotic syndrome (14). Our case was a 5-year-old girl without any evidences of thrombophilia, but only with a mild dehydration and fever. Extensive CSVT without underlying disorders is an unexpected event, which is rarely reported, hence it was a strong reason for publishing this case.

The most common signs and symptoms of CSVT are severe headache, seizure (focal or generalized), papilledema, decreased level of consciousness at presentation or coma (3, 4) like our case, but sometimes subarachnoid hemorrhage has been reported (15). In neonates the primary neurologic manifestations are seizures and diffuse neurologic signs (16). Pseudotumor cerebri as a complication of CSVT may happen which needs to be treated. Lumbar puncture and acetazolamide align with anticoagulant therapy are useful (17, 18).

Optic neuropathy is another complication of CSVT, thus frequent fundoscopic examination may be required (3). Brain CT scans, especially in neonates, may have false positive results.

Transfontanellar power Doppler ultrasonography is a useful method for monitoring neonatal sinovenous thrombosis, but desired technique for establishing the diagnosis in children is MRI with MRV (3).

But sometimes this method is prone to have artifacts, especially if deep venous infarction or cortical venous thrombosis is suspected. At these cases high-resolution CT venography or conventional digital subtraction angiography may be needed as a final arbiter. Treatment depends on the underlying disorder: supportive care, rehydration, parenteral antibiotic therapy, and in cases with head and neck infections even surgery (mastoidectomy, myringotomy, etc.).

Occasionally patients show intracranial hypertension as pseudotumor cerebri which may require serial lumber punctures and long term acetazolamide therapy, like our patient (19).

Administration of anticoagulants in the acute phase with either parenteral unfractionated heparin, subcutaneous low molecular weight heparin (LMWH), or warfarin in older infants and children is preferred (20).

This regimen is followed by a long term anticoagulant therapy (LMWH) for 3 to 6 months (19).

Several studies have demonstrated that anticoagulant therapy improves outcome due to mortality and morbidities. There are no practical outcomes on thrombolysis or surgical decompression in CSVT in children and even adults (3, 20). CSVT mortality is less than 10%, but neurologic impairments are present in 17% to 79 % of survivors. Coma is a predictor of death. Neurologic squeals are visual problems, hemiparesis, developmental delay and learning disabilities.

In older ages, the absence of parenchymal abnormality, anticoagulation, sigmoid and / or lateral sinus involvements are associated with a better outcome (3).

In this case although there was extensive cerebral sinovenous thrombosis, but early treatment (rehydration and anticoagulant therapy) resulted in a good outcome, and she had no neurological deficits after one month while was taking warfarin.

Because 10 – 20% of children with CSVT would have recurrent events, long follow up is very important. We could not follow up our case more than a month, and it was a week point in this report.

There have not been trials of strategies to prevent recurrences. Anticoagulants therapy should be taken into account for up to 6 months after the first event.
